# Nitrogen Addition Affects Ecosystem Carbon Exchange by Regulating Plant Community Assembly and Altering Soil Properties in an Alpine Meadow on the Qinghai–Tibetan Plateau

**DOI:** 10.3389/fpls.2022.900722

**Published:** 2022-06-13

**Authors:** Ling Han, Hasbagan Ganjurjav, Guozheng Hu, Jianshuang Wu, Yulong Yan, Luobu Danjiu, Shicheng He, Wendong Xie, Jun Yan, Qingzhu Gao

**Affiliations:** ^1^Institute of Environment and Sustainable Development in Agriculture, Chinese Academy of Agricultural Sciences, Beijing, China; ^2^National Agricultural Experimental Station for Agricultural Environment, Nagqu, China; ^3^China New Era Group Corporation, Beijing, China; ^4^Nagqu Grassland Station, Nagqu, China

**Keywords:** alpine meadow, nitrogen deposition, net ecosystem carbon exchange, soil available nitrogen, aboveground biomass, plant community

## Abstract

Nitrogen (N) deposition can affect the global ecosystem carbon balance. However, how plant community assembly regulates the ecosystem carbon exchange in response to the N deposition remains largely unclear, especially in alpine meadows. In this study, we conducted a manipulative experiment to examine the impacts of N (ammonium nitrate) addition on ecosystem carbon dioxide (CO_2_) exchange by changing the plant community assembly and soil properties at an alpine meadow site on the Qinghai–Tibetan Plateau from 2014 to 2018. The N-addition treatments were N0, N7, N20, and N40 (0, 7, 20, and 40 kg N ha^–1^year^–1^) during the plant growing season. The net ecosystem CO_2_ exchange (NEE), gross ecosystem productivity (GEP), and ecosystem respiration (ER) were measured by a static chamber method. Our results showed that the growing-season NEE, ER and GEP increased gradually over time with increasing N-addition rates. On average, the NEE increased significantly by 55.6 and 65.2% in N20 and N40, respectively (*p* < 0.05). Nitrogen addition also increased forage grass biomass (GB, including sedge and Gramineae) by 74.3 and 122.9% and forb biomass (FB) by 73.4 and 51.4% in N20 and N40, respectively (*p* < 0.05). There were positive correlations between CO_2_ fluxes (NEE and GEP) and GB (*p* < 0.01), and the ER was positively correlated with functional group biomass (GB and FB) and soil available N content (NO_3_^–^–N and NH_4_^+^–N) (*p* < 0.01). The N-induced shift in the plant community assembly was primarily responsible for the increase in NEE. The increase in GB mainly contributed to the N stimulation of NEE, and FB and the soil available N content had positive effects on ER in response to N addition. Our results highlight that the plant community assembly is critical in regulating the ecosystem carbon exchange response to the N deposition in alpine ecosystems.

## Introduction

Accelerating industrialization and increasing nitrogen (N) fertilizer application have substantially increased the N deposition in most areas of the earth (Canfield et al., 2010), which will make reducing greenhouse gas emissions harder in the coming decades (IPCC, 2018). The release and deposition of global atmospheric reactive N into the Earth’s surface is predicted to total 200 Tg N year^–1^ by 2050 (Galloway et al., 2008). Increasing the N deposition alters plant-community compositions and affects the biogeochemical cycling of terrestrial ecosystems (Simkin et al., 2016), particularly in N-limited grasslands (Canfield et al., 2010). Consequently, the N deposition can have many effects on the plant productivity and ecosystem carbon fluxes in grasslands (Niu et al., 2010; Zhang J. et al., 2020).

Net ecosystem carbon exchange (NEE), which is defined as the difference between gross ecosystem productivity (GEP) and ecosystem respiration (ER), is widely used to describe the changes in ecosystem carbon sinks and sources and is a vital function in the global carbon cycle (Mahecha et al., 2010; You et al., 2020). However, the effect of N addition on grassland NEE has been shown to be either positive (Niu et al., 2010) or non-significant (Kim and Henry, 2013) in different grassland ecosystems. Such inconsistent findings are likely due to different frequencies and concentrations of N addition (Cao et al., 2019) and the changes in climate factors (temperature and precipitation) (Niu et al., 2010; Yan et al., 2011). Additionally, the soil temperature, water availability, soil nutrient availability (Leff et al., 2015; Wang et al., 2020), and community composition (Xu et al., 2016) affect ecosystem carbon dioxide (CO_2_) fluxes under N addition.

The dynamic response of ecosystem carbon exchange to N addition is derived from shifts in species composition and colimitation with other abiotic resources (Jiang et al., 2012). A long-term N addition affects the plant biomass and community composition in grassland ecosystems by increasing soil N availability (Bai et al., 2008; Yang et al., 2012; Xu et al., 2014; Shen R. N. et al., 2022). For example, a long-term N addition could promote the functional groups of grass by negatively impacting forb in alpine grasslands (Zhang L. et al., 2020), which is an effect related to the different mycorrhizal distributions, N absorption amounts and utilization mechanisms between grasses and forbs (Wang et al., 2015; Kang et al., 2019). Chronic or excessive N addition can also reduce the species richness and diversity when soil N exceeds the saturation threshold (Xia and Wan, 2008; Li S. et al., 2019). Furthermore, many studies have shown that N addition can affect ER and NEE by stimulating plant growth and altering the plant community composition. For example, N addition stimulated NEE mainly by increasing the cover of dominant species in alpine meadows (Shen H. et al., 2022). Nitrogen-induced shifts in plant functional groups are considered to be an essential factor affecting carbon exchange in ecosystems (Niu et al., 2010; Fang et al., 2011; Chen et al., 2020), generally favoring a few nitrophilic plant species while suppressing the growth of many other species (Duprè et al., 2010; Jiang et al., 2012). In addition, studies showed that an increase in soil respiration was positively related to soil nutrient availability under N addition (Leff et al., 2015; Peng et al., 2015; Riggs et al., 2015).

Nitrogen deposition is a prominent global change driver on the Qinghai–Tibetan Plateau (QTP) (Liu et al., 2015) because the rapid development of the regional economy and the long-distance monsoon transport of atmospheric N deposition from South Asia have been related to the conspicuously increasing trend of the N deposition rate (Lü and Tian, 2007; Liu et al., 2015). Alpine meadows are one of the typical or dominant grassland ecosystem types on the QTP and are vital carbon pools in the “third pole” (Wang et al., 2014; Gao et al., 2016). Alpine meadows have long been affected by N and water deficiencies (Qiu, 2008; Liu et al., 2018; Dong et al., 2020), and their vegetation compositions and ecological processes are highly sensitive to the N deposition (Xu et al., 2018; Ma et al., 2019). The effects of N deposition on ecosystem CO_2_ fluxes and plant community assembly have been widely studied in this region. A study from the QTP found that the N deposition could shift plant species composition in favor of graminoids (Shen H. et al., 2022). The responses of the functional traits of alpine plants to the N deposition showed cascading effects from dominant species to functional groups and plant communities (Li et al., 2022). A long-term N addition can greatly reduce species richness (Li S. et al., 2019), and NEE may increase under N addition (Niu et al., 2009, 2010). Multigradient N-addition experiments have shown that the GEP revealed a non-linear increasing trend with increasing the N deposition rates (Tian et al., 2015). In addition, some researchers have highlighted that the ecosystem CO_2_ fluxes were affected by plant productivity (Bai et al., 2008; Tian et al., 2015). However, how to achieve the combination of functional groups and promote ecosystem carbon fluxes under the N deposition in alpine meadows remains unclear. Based on the above studies, we hypothesized that N addition could stimulate ecosystem CO_2_ fluxes *via* increasing sedge and forb biomass, thereby affecting the responses of the carbon sink function in alpine meadows.

A 5-year (2014–2018) field-manipulation experiment with four N deposition rates (i.e., N0, N7, N20, and N40) was conducted in an alpine meadow on the QTP. Our study aimed to (1) determine how plant functional groups and ecosystem CO_2_ fluxes respond to increasing N deposition and (2) clarify how the associated changes in the plant community assembly affect the responses of ecosystem CO_2_ fluxes to N addition.

## Materials and Methods

### Study Site

The research area is located at the Nagqu National Agricultural Experimental Station for the Agricultural Environment, Tibet Autonomous Region, China (31.441°N, 92.017°E) at an elevation of 4,500 m. The mean annual temperature and precipitation are −1.2°C and 431.7 mm, respectively. The main functional groups are sedge, *Kobresia pygmaea*, *Carex moorcroftii*, Gramineae, *Poa pratensis* and forbs (mainly including *Potentilla acaulis* and *Oxytropis ochrocephala*) (Zhou et al., 2021). The soil is clay silt and is classified as alpine meadow soil. The soil bulk density is 1.01 g cm^–3^. The experimental field was grazed by yak (*Bos grunniens*) every summer before 2010, after which the study area was fenced off, and grazing and mowing were prohibited throughout the experiment (Ganjurjav et al., 2020). The growing season is from May to September when the average temperature is above 0°C, and the precipitation during this time accounts for more than 90% of the total precipitation. The precipitation was lower during the middle of the growing season in 2015 and 2017, and the temperature showed an undulating increasing trend during the growing season in the time period 2014-2018 (Supplementary Figure 1).

### Experimental Design

The total dry and wet N deposition rate was approximately 7 kg N ha^–1^.year^–1^ (6.96–7.55 kg N ha^–1^.year^–1^) in the Tibet Autonomous Region (Lü and Tian, 2007; Liu et al., 2013). Therefore, we set N-addition rates of approximately 1, 3, and 6 times 7 kg N ha^–1^.year^–1^ to simulate the N deposition effects on ecosystem carbon exchange in the study area. We initiated a 5-year (from 2014 to 2018) N deposition experiment in an alpine meadow using a randomized block design with 16 experimental plots (each plot of size 3 m × 3 m) separated by a 2-m buffer. One control (unfertilized, N0) and three N addition treatments termed N7, N20, and N40 (0, 7, 20, and 40 kg N ha^–1^ year^–1^, respectively) were randomly assigned to plots. Four replicate plots were selected for use in our study. Ammonium nitrate (NH_4_NO_3_) was applied to simulate atmospheric N deposition, and each plot was sprayed with a solution of NH_4_NO_3_ dissolved in 5 L water 4 times per year (early in each month from May to August). The same volume of water was sprayed on the control plots to avoid an impact from added water. The total annual volume of water applied to each plot was 40 L, which was equivalent to 1% of the local annual precipitation (Yan et al., 2018).

#### Plant Community Assembly

The community characteristics were surveyed twice a month during the growing season from 2014 to 2018. The community characteristic data during the peak growing season (late July or early August) were used in this study. A small quadrat (of size 0.5 m × 0.5 m) in each plot was selected for the determination of vegetation characteristics using sampling and visual methods. First, we recorded and measured the numbers and heights of all plant species. The heights of five individuals of each species were measured, and the mean value was used to represent the species height. Second, we estimated the total community coverage (total cover) and the coverage of each species (percentage coverage) using a visual method. Third, the aboveground biomass of different functional groups was determined by non-destructive measurement (Ganjurjav et al., 2020). The non-destructive measurement method included establishing a regression equation of plant height, coverage, and biomass in the vicinity of the sample plot and estimating the plant biomass in the plot using the developed equation (see Supplementary Table 1). The species richness index was equal to the number of species. Species dominance was evaluated based on the importance value index (*P*), which was computed as follows:


(1) $$P_{\text{i}}=\left(FC_{i}+RF_{i}\right)/2$$

where *RC*_*i*_ and *RF*_*i*_ are the relative cover (equal to the ratio of the species coverage to the total coverage of all species) and relative height (equal to the ratio of the species height to the total height of all species), respectively (Sun et al., 2021).

The plant community diversity was computed using the Shannon–Wiener index as follows:


(2) H=′-ΣPilnPi

where *P*_*i*_ represents the importance value index of the *i*th species (Magurran, 1988).

#### Ecosystem Carbon Dioxide Fluxes

A portable photosynthesis system (Li-6400; LI-COR Inc., Lincoln, NE, United States) and the transparent chamber method were used to measure the carbon exchange 1–3 times a month during the growing season from 2014 to 2018. We carried out these field measurements at 10:00–12:00 local time on sunny days. First, on the inner side of the top of each transparent polyethylene chamber (of size 0.3 m × 0.3 m × 0.4 m), a fan was installed to mix the gas inside the chamber during the measurement. Second, a transparent polyethylene chamber was placed in each quadrat to measure NEE for 90 s. Then, we removed the chamber to ensure that the air humidity and CO_2_ reached at ambient levels. Finally, we placed the chamber back in each quadrat, covered it with an opaque shade cloth, and measured ER for 90 s. Gross ecosystem productivity was calculated using the NEE and ER values. For more information on this methodology, see Ganjurjav et al. (2015).

#### Soil Properties

The soil samples were collected from 0 to 10 cm soil layer in each plot using an auger in mid-August every year. The soil available N content (NH_4_^+^–N and NO_3_^–^–N) was extracted with a 2 mol L^–1^ KCl solution and determined with a continuous flow spectrophotometer (FIAstar 5000 Analyzer; Foss Tecator, Hillerød, Denmark). The soil microbial biomass C (MBC) and N (MBN) were measured using the chloroform fumigation extraction method (Brookes et al., 1985; Fu et al., 2012). Fumigated and unfumigated soil samples were extracted with 100 ml 0.5 mol L^–1^ K_2_SO_4_ and filtered with a 0.45-μm membrane. The extractable organic carbon and total N were determined by an automatic carbon analyzer (Phoenix 8000) and flow injection nitrogen analyzer (FIAStar5000, FOSS Inc), respectively. Extractable carbon and N were converted to MBC and MBN using a conversion coefficient of 0.45 for both measurements (Fu et al., 2012).

### Data Analysis

The annual maximum ecosystem carbon exchange value (obtained between late July and early August in each year) was used to present the annual variation in NEE, GEP, and ER during the growing season. We log_10_-transformed all variables before performing the data analysis to stabilize the residual variances. One-way ANOVA and *post hoc* tests were used to analyze significant differences in ecosystem CO_2_ fluxes, soil properties, community diversity, and functional group biomass among the N0, N7, N20, and N40 plots over 5 years. The functional group biomass included forage grass and forb biomass (GB and FB, respectively), and forage grass was defined as the combination of sedge and Gramineae (Huang et al., 2020). Differences were considered significant when *p* < 0.05. We also applied repeated-measures analysis of variance (RMANOVA) to analyze the individual and interactive influences of year and N addition on CO_2_ fluxes (i.e., NEE, ER, and GEP), soil properties, community diversity, and functional group biomass. Pearson correlation analysis was employed to analyze the relationships between CO_2_ fluxes (NEE, GEP and ER) and soil properties, community diversity, and functional group biomass over 5 years. Meanwhile, to determine the long-term effect of N addition, we calculated the response ratios under the treatments (N7, N20, and N40) relative to the control (N0) to examine the changes in biomass, and the change in carbon flux was defined as the difference between the value under each N addition treatment and that under N0 treatment. All analyses were carried out using SPSS (SPSS for Windows, version 22.0).

We established *a priori* structural equation modeling (SEM; IBM SPSS Amos 24) based on correlation analyses between the CO_2_ fluxes (NEE and ER) and GB, FB and soil properties (Supplementary Figure 4 and Supplementary Table 4). Next, we explored how N addition affected NEE and ER by changing soil properties and functional group biomass. A multivariate index representing the soil available N content was created by principal component analysis (PCA) of the soil NO_3_^–^–N and NH_4_^+^–N contents. The first principal component explained 66.5% of the total variance and was used in the SEM analysis. First, we hypothesized that N addition would increase the soil properties, and alter the plant community assembly by increasing soil nutrient availability (Bai et al., 2008; Yang et al., 2012; Xu et al., 2014), which would then affect the ER and NEE responses to N addition (Duprè et al., 2010; Jiang et al., 2012). We optimized the prior model based on the Chi-squared (χ^2^) statistic (*p*>0.05), root mean square error of approximation (RMSEA)<0.05, comparative fit index (CFI) and Tucker–Lewis index (TLI)>0.95, and the lowest Akaike information criterion (AIC) value (Wei et al., 2013).

## Results

### Changes in Soil Chemical Properties

**FIGURE 1:**
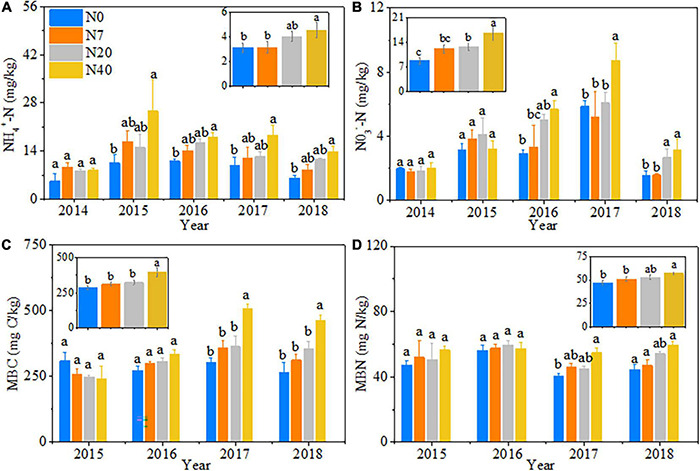
Patterns in soil properties with increasing N addition from 2014 to 2018. N0–N40: expressed as control, 0, 7, 20, and 40 kg N ha^–1^year^–1^. **(A)** NH_4_^+^–N, soil ammonium nitrogen; **(B)** NO_3_^–^–N, soil nitrate–nitrogen; **(C)** MBC, soil microbial biomass carbon; **(D)** MBN, soil microbial biomass nitrogen. The error bars represent the standard error (SE). Different lowercase letters indicate significant differences (*p* < 0.05) among different N-addition levels (hereinafter inclusive). Some results are cited from Yan (2019).

Nitrogen addition had significant effects on soil properties (Table 1, *p* < 0.05). Compared to the N0 plot, the soil NO_3_^–^–N, NH_4_^+^–N, MBC, and MBN contents increased almost exclusively in the N40 treatment (Figure 1). On average, the soil NH_4_^+^-N, MBC and MBN contents increased by 44.3, 38.5, and 21.0% in N40, respectively, and the soil NO_3_^–^–N content increased by 45.7 and 88.0% in N20 and N40, respectively (*p* < 0.05). The soil properties changed significantly during the 5 studied years (Table 1, *p* < 0.05). Specifically, in N40, the soil NH_4_^+^–N content was 153.6, 63.4, 86.9, and 124.9% higher than that in N0 in 2015–2018 (Figure 1A). Additionally, the soil MBC content was 67.7 and 74.4% while MBN content was 35.7 and 33.5% higher than that in N0 in 2017–2018, respectively (Figures 1C,D). The soil NO_3_^–^–N content was also 93.5, 49.8, and 99.4% higher than that in N0 in 2016–2018, respectively (Figure 1B). In N20, the soil NO_3_^–^–N content was 71.1 and 69.6% higher than that in N0 in 2016 and 2018, respectively (Figure 1B).

**TABLE 1:** Results of repeated measures analysis of variance (RMANOVA) on the individual and interactive effects of N addition (N), year on soil properties.

Factors		NO_3_^–^–N (mg kg^–1^)		NO_3_^–^–N (mg kg^–1^)			MBC (mg C kg^–1^)		MBN (mg N kg^–1^)	
	*df*	*F*	*p*	*F*	*p*	*df*	*F*	*p*	*F*	*p*
Year	4	20.61	**<0.001**	4.33	**0.009**	3	15.34	**<0.001**	6.29	**0.001**
Nitrogen	3	4.65	**0.007**	8.22	**<0.001**	3	10.91	**0.001**	4.70	**0.006**
Year × Nitrogen	12	1.05	0.42	0.43	0.91	9	4.14	**0.001**	0.81	0.61

*The numbers in bold express significant effects (p < 0.05). NH_4_^+^–N, soil ammonium nitrogen; NO_3_^–^–N, soil nitrate-nitrogen; MBC, soil microbial biomass carbon; MBN, soil microbial biomass nitrogen.*

### Changes in Forb and Forage Grass Biomass and Aboveground Biomass

**FIGURE 2:**
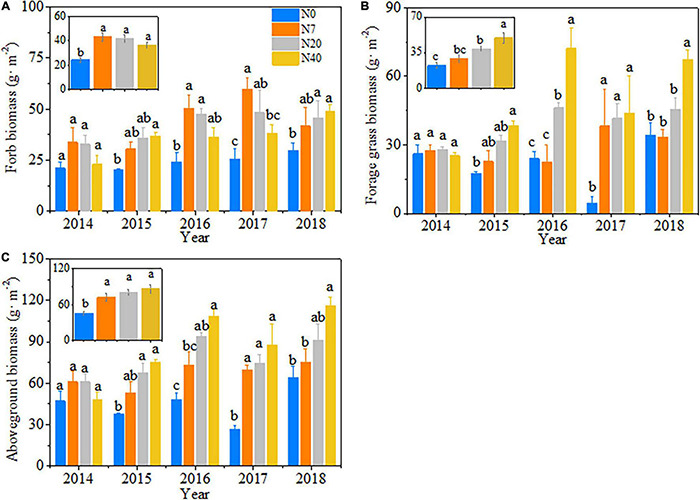
Patterns in **(A)** forb biomass, **(B)** forage grass biomass, and **(C)** aboveground biomass with increasing N addition from 2014 to 2018. The error bars represent the standard error (SE). N0–N40: expressed as control, 0, 7, 20, and 40 kg N ha^–1^year^–1^. Different lowercase letters indicate the significant differences (*p* < 0.05) among different N-addition levels.

**FIGURE 3:**
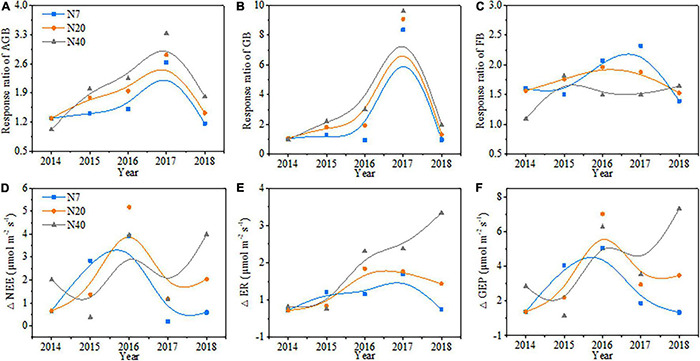
Response ratio of **(A)** aboveground biomass (AGB), **(B)** forage grass biomass (GB) and **(C)** forb biomass (FB) and changes in **(D)** net ecosystem CO_2_ exchange (NEE), **(E)** ecosystem respiration (ER), and **(F)** gross ecosystem productivity (GEP) from 2014 to 2018.

The variations in aboveground biomass and forage grass and forb biomass mainly depended on the N-addition level, and differences were also observed among the 5 studied years (Table 2, *p* < 0.05). The aboveground biomass and forage grass biomass mostly showed gradual increasing trends (Figures 2B,C), and forb biomass mostly showed a unimodal trend with N addition across the 5 studied years (Figure 2A). On average, the aboveground biomass increased by 59.1, 78.1, and 90.1% in N7, N20, and N40, respectively; forage grass biomass increased by 74.3 and 122.9% in N20 and N40, respectively; and forb biomass increased by 78.1, 73.4, and 51.4% in N7, N20, and N40, respectively (*p* < 0.05). The effects of N addition on AGB and GB followed a unimodal temporal trend, with the maximum response peak in 2017 (Figures 3A,B). The response ratios of forb biomass to N addition showed a different temporal trend, with N addition increasing FB in most years (Figure 3C).

**TABLE 2:** Results of repeated measures analysis of variance (RMANOVA) on the individual and interactive effects of N addition (N), and year on CO_2_ fluxes (GEP, ER and NEE), biomass (AGB, GB and FB) and diversity indices.

Factors		NEE (μ mol m^–2^s^–1^)		ER (μ mol m^–2^s^–1^)		GEP (μ mol m^–2^s^–1^)		AGB (g.m^–2^)		GB (g.m^–2^)		FB (g.m^–2^)		Species richness		Shannon–Wiener index	
	*df*	*F*	*p*	*F*	*p*	*F*	*p*	*F*	*p*	*F*	*P*	*F*	*P*	*F*	*P*	*F*	*p*
Year	4	20.85	**<0.001**	27.07	**<0.001**	17.45	**<0.001**	15.57	**<0.001**	6.29	**<0.001**	5.77	**0.001**	4.18	**0.005**	8.01	**<0.001**
Nitrogen	3	7.50	**<0.001**	4.81	**0.005**	8.18	**<0.001**	32.31	**<0.001**	16.61	**<0.001**	13.45	**<0.001**	12.74	**<0.001**	12.70	**<0.001**
Year × Nitrogen	12	1.93	**0.048**	0.69	0.76	1.37	0.21	2.75	**0.005**	2.62	**0.007**	1.34	0.22	2.17	**0.03**	1.18	0.32

*The numbers in bold express significant effects (p < 0.05). GEP, gross ecosystem productivity; ER, ecosystem respiration; NEE, net ecosystem carbon exchange; AGB, aboveground biomass; GB, forage grass biomass; FB, forb biomass.*

### Changes in Plant Community Diversity

**FIGURE 4:**
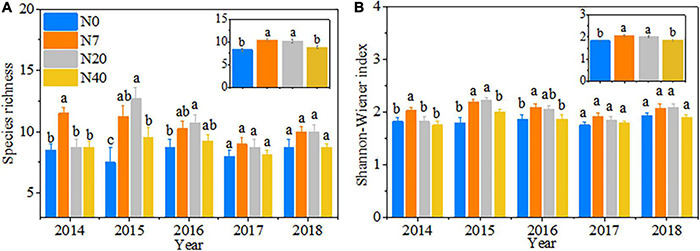
Patterns in **(A)** species richness and **(B)** Shannon–Wiener index from 2014 to 2018. The error bars represent the standard error (SE). N0–N40: expressed as control, 0, 7, 20, and 40 N ha^–1^year^–1^. Different lowercase letters indicate significant differences (*p* < 0.05) among different N-addition levels.

Species richness was prominently affected by the year, N addition, and their interactions (Table 2, *p* < 0.05). The variations in the Shannon–Wiener index mainly depended on the N-addition level (*p* < 0.05). The species richness and the Shannon–Wiener index first increased and then decreased over time with increasing N-addition rates, peaking between the N7 and N20 plots (Figures 4A,B). The differences were also observed among the 5 studied years (Table 2, *p* < 0.05). In 2014, the species richness and Shannon–Wiener index in N7 were significantly higher than those in the other three plots (Figures 4A,B, *p* < 0.05). In 2015, the species richness and Shannon–Wiener index in N7 and N20 were significantly higher than those in N0 and N40 (Figures 4A,B, *p* < 0.05). In 2016, the maximum species richness and Shannon–Wiener index values were found in N20 and N7, respectively (Figures 4A,B, *p* < 0.05).

### Changes in Ecosystem Carbon Dioxide Exchange With Increasing Nitrogen Addition

**FIGURE 5:**
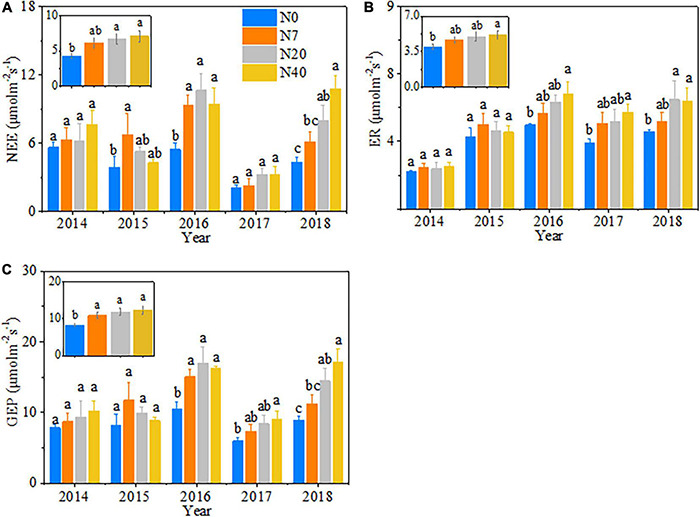
The changes in **(A)** net ecosystem carbon exchange (NEE), **(B)** ecosystem respiration (ER), and **(C)** gross ecosystem productivity (GEP) with increasing N addition from 2014 to 2018. N0–N40: expressed as control, 0, 7, 20, and 40 kg N ha^–1^year^–1^. The error bars represent the standard error (SE). Different lowercase letters indicate significant differences (*p* < 0.05) among different N-addition levels.

The variations in ecosystem CO_2_ fluxes (i.e., NEE, ER, and GEP) mainly depended on N-addition level (*p* < 0.05), and significant differences in NEE were observed among the 5 studied years (Table 2, *p* < 0.05). Generally, NEE, ER, and GEP increased gradually over time with increasing N addition rates and showed an interannual fluctuating trend across the 5 years (Figures 3D–F). On average, NEE increased by 55.6 and 65.2% in N20 and N40, respectively, ER increased by 30.3% in N40, and GEP increased by 30.7, 42.8, and 48.4% in N7, N20, and N40, respectively (*p* < 0.05). In 2015, NEE, ER, and GEP showed a unimodal trend with the N-addition level, and in N7, NEE was 72.8% higher than that in N0 (Figure 5A, *P* < 0.05). In 2014 and 2017, the response of NEE to N addition showed a gradual increase but did not significantly differ from that of N0 (Figure 5A, *P* > 0.05). In 2016, N addition (N7, N20, and N40) significantly increased NEE, which reached 71.6, 94.9, and 72.5%, respectively (*p* < 0.05). In 2018, NEE significantly increased by 83.7 and 152.2% in N20 and N40, respectively (*p* < 0.05). Meanwhile, ER gradually increased in 2016–2018 with increasing N-addition levels. Specifically, ER significantly increased by 36.5 and 48.2% at N40 in 2016 and 2017, respectively (*p* < 0.05), and in 2018, ER increased significantly by 42.0 and 39.2% in N20 and N40, respectively (Figure 5B, *P* < 0.05). In addition, the response of GEP to N addition showed a change pattern similar to that of NEE (Figure 5C). In 2016, the GEP increased significantly by 43.6, 62.3, and 55.1% in N7, N20, and N40, respectively (*p* < 0.05). In 2017, the GEP increased by 51.0% in N40 (*p* < 0.05). In 2018, the GEP increased by 62.3 and 92.1% in N20 and N40, respectively (*p* < 0.05). The seasonal variations in CO_2_ fluxes showed that the maximum values all appeared between late July and early August in each year (Supplementary Figure 5).

### Plant Community Assembly and Soil Property Effects on Carbon Dioxide Fluxes

There were significant positive correlations between AGB and GB and NEE, GEP, and ER (*p* < 0.01). Both ER and GEP were positively affected by FB (*p* < 0.05). In addition, NEE was positively correlated with the soil MBN content (*p* < 0.01); ER was positively correlated with the soil properties (NO_3_^–^–N, NH_4_^+^–N, MBC, and MBN) and Shannon–Wiener index (*p* < 0.05); GEP was positively correlated with the soil MBN content and Shannon–Wiener index (*p* < 0.01).

**FIGURE 6:**
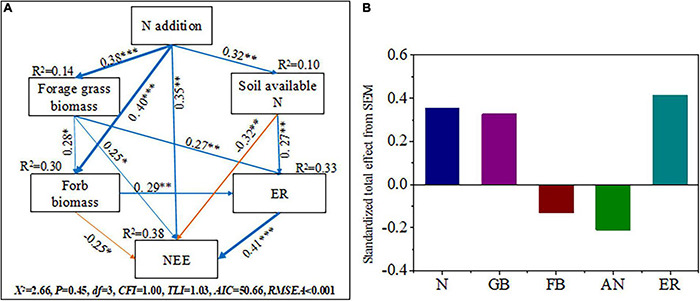
Structural equation models reveal direct and indirect influences of soil available N, forage grass biomass and forb biomass on the CO_2_ fluxes (NEE: net ecosystem CO_2_ exchange, ER: ecosystem respiration) to N addition **(A)** and **(B)**. N, N addition; GB, forage grass biomass; FB, forb biomass; AN, soil available N. Single-arrowed pathways indicate the directional effect between variables. The values associated with pathways are the standardized path coefficients. The *R*^2^-values are given for soil available N, forage grass biomass, forb biomass, ER, and NEE, indicating the variance explained by the model (*R*^2^). The fitness statistics, Chi-squared (χ^2^), degrees of freedom (*df*), P-value and root mean square error of approximation (RMSER), Tucker–Lewis index (TLI), comparative fit index (CFI) are given for the fitness of the model. The χ^2^-test with *p* > 0.05 and the RMSER ≤ 0.05 indicates that the model is acceptable. The width of the arrows indicates the strength of the relationships. Blue arrows indicate significant positive relationships and orange arrows indicate significant negative relationships. The numbers on the line indicate standardized path coefficients. Stars indicate significant correlations. *p < 0.05, **p < 0.01, ***P < 0.001. The data shown here include all data collected across the treatments and years. See Supplementary Figure 4 and Supplementary Table 4 for more details of the *a priori* model.

Structural equation models adequately fitted the data describing the interaction pathways among the soil available N (AN), forage grass biomass (GB), forb biomass (FB), and ecosystem CO_2_ fluxes (NEE and ER) in response to N addition (Figures 6A,B). The models explained 10.0, 14.4, 30.2, 32.7, and 38.2% of the variations in soil available N, forage grass biomass, forb biomass, ER and NEE, respectively (Figure 6A). The results of SEM analysis illustrated that N addition increased NEE indirectly through soil available N, forage grass biomass, and forb biomass and ER (0.35). Forage grass biomass (0.341) and ER (0.41) were positively correlated with NEE, while soil available N (-0.32) and forb biomass (-0.25) were negatively correlated with NEE (*p* < 0.05). Nitrogen addition indirectly increased ER *via* soil available N, forage grass biomass and forb biomass (0.33); ER was positively correlated with soil available N (0.27), forage grass biomass (0.27), and forb biomass (0.29). In addition, N addition significantly increased soil available N (0.32, *p* < 0.01), forage grass biomass (0.38, *p* < 0.001), forb biomass (0.40, *p* < 0.001), and NEE (*p* < 0.01, 0.35). Forage grass biomass was positively correlated with forb biomass (*p* < 0.05).

## Discussion

### Effects of Nitrogen Addition on Functional Group Biomass and Plant Community Assembly

The soils of the most grassland ecosystems are characterized as N-poor, which affects the plant community composition and biomass accumulation (Wang et al., 2015; Shen R. N. et al., 2022). Our results found that AGB, GB, and FB increased significantly with N-addition levels under seasonal N application during the growing season (Figure 2). Our results confirm the previous studies indicating that N addition significantly enhances aboveground biomass by providing a large amount of N required for plant growth (Bai et al., 2010; Tian et al., 2015). Simulated N deposition has many positive impacts on plants when N application occurs more evenly throughout the year instead of at a single time (Cao et al., 2019). We also found that soil available N (NO_3_^–^–N and NH_4_^+^–N) increased significantly at higher N levels (Figures 1A,B), providing sufficient nutrients for the plant community growth (Han et al., 2019). There were significant correlations between AGB and GB and the soil NH_4_^+^–N content (Table 3) which also supported the above results. Meanwhile, consistent with previous studies (Xu et al., 2014; Armstrong et al., 2015), we found that AGB and functional group biomass largely depended on precipitation and temperature during the growing season (Supplementary Figure 2). In particular, AGB and GB increased significantly in 2017 (Figures 3A,B), possibly because the higher precipitation (114.05 mm) in the early growing season (Supplementary Figure 2) improved the plant biomass production by increasing the uptake and utilization of N. Moreover, these results indicated that the synergistic effects of climate and soil factors promoted grassland productivity (Shen R. N. et al., 2022).

**TABLE 3:** Pearson’s correlations between soil properties and plant community characteristics and CO_2_ fluxes during the growing season from 2014 to 2018.

	NEE (μ mol m^–2^s^–1^)	ER (μ mol m^–2^s^–1^)	GEP (μ mol m^–2^s^–1^)	AGB (g.m^–2^)	GB (g.m^–2^)	FB (g.m^–2^)	NO_3_^–^-N (mg kg^–1^)	NH_4_^+^–N (mg kg^–1^)	MBC (mg C kg^–1^)	MBN (mg N kg^–1^)	Shannon–Wiener index
NEE (μmol m^–2^s^–1^)	1										
ER (μmol m^–2^s^–1^)	0.39**	1									
GEP (μmol m^–2^s^–1^)	0.93**	0.69**	1								
AGB (g.m^–2^)	0.35**	0.53**	0.48**	1							
GB (g.m^–2^)	0.39**	0.45**	0.48**	0.89**	1						
FB (g.m^–2^)	0.15	0.45**	0.30**	0.78**	0.41**	1					
NO_3_^–^-N (mg kg^–1^)	–0.20	0.32**	–0.03	0.17	0.14	0.20	1				
NH_4_^+^-N (mg kg^–1^)	0.12	0.32**	0.21	0.29**	0.28*	0.17	0.35**	1			
MBC (mg C kg^–1^)	0.09	0.30*	0.16	0.48**	0.47**	0.28*	0.33*	0.05	1		
MBN (mg N kg^–1^)	0.48**	0.32*	0.48**	0.37**	0.39**	0.13	–0.11	0.26*	0.15	1	
Shannon–Wiener index	0.17	0.25*	0.24*	0.22	0.01	0.39**	–0.12	0.16	−0.42**	0.14	1
Species richness	0.18	0.10	0.18	0.16	0.04	0.26*	–0.06	0.22*	−0.39**	0.14	0.89**

NEE, net ecosystem exchange; ER, ecosystem respiration; GEP, gross ecosystem productivity; AGB, aboveground biomass; GB, forage grass biomass; FB, forb biomass; MBC, soil microbial carbon; MBN, soil microbial nitrogen. *Correlation is significant at the 0.05 level. **Correlation is significant at the 0.01 level.

There were differences in the sensitivity of different functional groups to N addition because of the difference in the N utilization capacity of plant roots and leaves (Kang et al., 2019). Some previous studies in alpine (Bassin et al., 2007) and subalpine grasslands (Soudzilovskaia and Onipchenko, 2005) reported that sedges were characterized by high respiration efficiency and growth rates (Aerts, 1999; Shane et al., 2006; Mcclean et al., 2011), which will make sedges have a higher resource competitiveness than the other functional groups in alpine meadow ecosystems. We can partially support our hypothesis that a higher N addition could change the plant community assembly through shifts in composition toward forage grass-dominated communities. Sedge and Gramineae dominance over forb increased at high N (N40) level (Supplementary Figure 3), especially during a dry year (in 2015) (Supplementary Figure 1), which is consistent with the results of a previous study (Shen et al., 2019). This result may be because the whisker root system of forage grass (sedge and Gramineae) mainly distributed in the surface soil in alpine meadows and can access soil available N and water resources more easily than the root system of forb. Gramineae species such as *P. pratensis* are in the upper part of meadow canopy and more competitive for light and soil nutrients (Hautier et al., 2009). Significant increases in sedge coverage and Gramineae height also limited the competitiveness of forb for light (Supplementary Tables 2,3). Thus, N deposition is likely to enhance the dominant position of forage grass over forb in alpine meadows. Moreover, similar to the previous studies (Niu et al., 2010; Li S. et al., 2019), we found that plant diversity (species richness and Shannon–Wiener index) had a non-linear response trend, and this facilitating effect decreased with increasing N-addition rates (Figure 4). Nitrogen deposition is an ongoing process, and high N input may lead to a saturation response. The limiting resource for plants may be the availability of water, light, and other resources instead of N (Niu et al., 2010). The rapid growth of N-loving plants (forage grass) reduces the light transmittance of vegetation and causes the loss of plant diversity due to plant light competition (Hautier et al., 2009).

### Effects of Nitrogen Addition on Ecosystem Carbon Dioxide Fluxes

A long-term N addition will influence carbon cycling (Simkin et al., 2016). Inner Mongolian grassland experiments found that the responses of CO_2_ fluxes all exhibited non-linear patterns with increasing N-addition rates (Niu et al., 2010; Tian et al., 2015). In contrast to the previous results, our results showed that the response of ecosystem CO_2_ fluxes (NEE, GEP, and ER) showed an increasing pattern with increasing N addition over the 5 years (Figures 5A–C). This pattern may illustrate that N and water availability are relatively lower in alpine meadows than in alpine steppes (Li S. et al., 2019), and alpine meadow ecological processes are highly sensitive to the N deposition during the growing season (Liu et al., 2015; Ma et al., 2019). Meanwhile, the relative contributions of GEP and ER significantly increased NEE because the response of GEP to cumulative N addition was more sensitive than that of ER (Figure 5). This result is because GEP is related only to plant photosynthesis, whereas ER is also affected by microorganisms and animals in the soil (Niu et al., 2009). There were significant correlations between ER and soil factors (MBC and MBN) (Table 3), possibly as a result of the increased availability of soil microbial respiration. A global meta-analysis by Zhou et al. (2017) also suggested that N rates less than 100 kg N ha^–1^year^–1^ stimulate microbial growth. Similar to a previous study (Tian et al., 2015), we demonstrated that the precipitation pattern affects the peak ecosystem carbon fluxes (Supplementary Figure 2); thus, NEE exhibited interannual differences (Figures 3D–F and Table 2). For example, the response of NEE to N addition was lower in 2017 than in other years, which may have been because lower precipitation amounts during the growing season (Supplementary Figure 1) lead to low plant photosynthetic rates (Niu et al., 2010). This phenomenon may also be because the increase in productivity was offset by boosting respiration consumption, which was also detected in temperate steppe (Niu et al., 2010) and Irish pasture systems (Peichl et al., 2011).

### Plant Community Assembly and Soil Properties Regulate Ecosystem Carbon Dioxide Fluxes Under Nitrogen Addition

**FIGURE 7:**
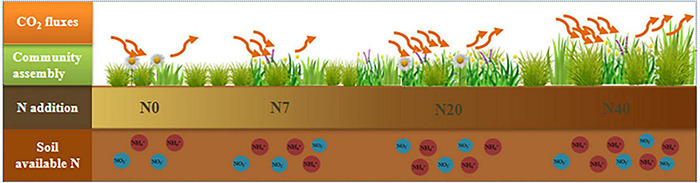
Conceptual diagram of the response of ecosystem CO_2_ fluxes (NEE, ER, and GEP) to regulation by the plant community assembly and soil properties under N addition (N0, N7, N20, and N40). Blue and red circles represent the soil available N content (NO_3_^–^–N and NH_4_^+^–N), respectively. The downward arrow is gross ecosystem productivity (GEP), and the upward arrow is ecosystem respiration (ER). Net ecosystem carbon exchange is defined as the difference between GEP and ER.

Our study provided evidence that the effects of N addition on ecosystem CO_2_ exchange are regulated through soil available N and the plant community assembly (Figures 6, 7). Kim and Henry (2013) found that the net influx was observed during periods of peak aboveground biomass. Our findings confirmed the previous conclusion that plant productivity plays a major role in adjusting the responses of ecosystem CO_2_ fluxes to N addition (Niu et al., 2009). Nitrogen addition alleviates soil nutrient constraints by increasing the soil available N at higher N-addition level (Figure 1 and Table 1); thus, promoting a rapid vegetation growth and biomass accumulation (Bai et al., 2008; Yang et al., 2012; Xu et al., 2014; Shen R. N. et al., 2022). Higher productivity provides more substrates and increases the leaf area index and photosynthetic units associated with photosynthesis production (Bassin et al., 2007; Wang et al., 2015).

According to our hypothesis (Supplementary Figure 4), the plant community assembly would positively regulate the responses of ecosystem CO_2_ fluxes to N addition (Figure 7). Nitrogen-stimulated forage grass could effectively promote NEE and ER because forage grass is construction species in the study area, and GB is significantly positive correlated with AGB and GEP (Figure 3, *p* < 0.05). Litter decomposition from forage grass roots and leaves continuously provides many carbon and N sources to the soil microbial community, which boosts soil respiration and NEE (Mcclean et al., 2011). Additionally, the positive response of ER to N addition depended on the increase in FB (Figure 6). The increase in forb coverage contributed to the higher levels of CO_2_ uptake (Supplementary Table 3, *p* < 0.05) because the larger leaf spread area of forb was accompanied by the higher leaf breathing capacity (Ammann et al., 2007). Therefore, the N-induced shift in the plant community assembly toward forage grass-dominated community affected the formation of carbon sinks in alpine meadows. In addition, the changes in soil available N also affected ER because N addition may increase soil available resources and in turn increase ER by increasing heterotrophic respiration in the soil (Leff et al., 2015; Riggs et al., 2015), and this finding was similar to that of a previous study on the QTP (Peng et al., 2015). Nitrogen addition will increase the chlorophyll concentration in the different functional groups, which might be another reason for the increases in NEE and ER (Yan et al., 2014). However, we found negative effects of soil available N on NEE possibly because the soil acidification induced by excessive N addition resulted in a decrease in community diversity; thus, reducing the soil carbon sink capacity (Tian et al., 2015; Li J. et al., 2019).

### Implications and Limitations

Consequently, our study showed that the plant community assembly significantly affected the response of the ecosystem CO_2_ fluxes to N deposition, thus promoting the grassland function as a carbon sink. Our results emphasized that the functional group (forage grass) was a more important regulator in promoting NEE and ER responses to the N deposition than soil available N in the alpine meadow (Figure 6B). However, we did not measure the changes in soil microbial activity in this experiment and could not explore the relationships between the plant community composition and microbial community composition (Hodge and Storer, 2015) or their responses to N addition. Future studies need to explore how these factors regulate the response of NEE to the N deposition and the soil carbon stability maintenance mechanism in alpine meadows.

## Conclusion

In an alpine meadow on the QTP, the ecosystem CO_2_ fluxes responded positively to N addition, with a higher increase in GEP than in ER, and N addition ultimately increased the net carbon uptake (measured as NEE) during the 5-year experiment. The forage grass and forb biomass all showed positive feedback to N addition because soil available N significantly increased with N addition. The plant community assembly changed in favor of forage grass under higher N addition. Nitrogen addition stimulated the ecosystem CO_2_ fluxes by regulating all functional group biomass and soil available N in the alpine meadow. Specifically, forage grass and forb had different regulatory effects on the response of CO_2_ fluxes to N deposition, and the increase in forage grass biomass mainly contributed to the N stimulation of NEE. Our study highlights the significant role of the plant community assembly in regulating NEE and ER under N addition. However, we found that excessive N addition reduced species diversity. Therefore, more attention should be given to the impacts of N addition on alpine meadows. Future works must evaluate whether changing the community composition and decreasing plant diversity will impair the long-term carbon sink functions of sensitive alpine meadows.

## Data Availability Statement

The original contributions presented in the study are included in the article/Supplementary Material, further inquiries can be directed to the corresponding author.

## Author Contributions

QG designed the research. YY, HG, GH, LD, SH, WX, and JY performed the experiments. LH analyzed the data and wrote the manuscript. LH, HG, GH, and JW interpreted the results and proofread the text. All authors contributed to this work and approved the final manuscript before submission.

## Conflict of Interest

YY was employed by China New Era Group Corporation. The remaining authors declare that the research was conducted in the absence of any commercial or financial relationships that could be construed as a potential conflict of interest.

## Publisher’s Note

All claims expressed in this article are solely those of the authors and do not necessarily represent those of their affiliated organizations, or those of the publisher, the editors and the reviewers. Any product that may be evaluated in this article, or claim that may be made by its manufacturer, is not guaranteed or endorsed by the publisher.
