# Supplementary material for: Nitrogen Addition Affects Ecosystem Carbon Exchange by Regulating Plant Community Assembly and Altering Soil Properties in an Alpine Meadow on the Qinghai–Tibetan Plateau
Source: Front Plant Sci. 2022 Jun 13;13:900722. doi: 10.3389/fpls.2022.900722 (PMC9234307; doi:10.3389/fpls.2022.900722)
Supplement: Supplementary file 1 [file Data_Sheet_1.docx]

**Supplements**

**Fig. S1. Changes in air temperature and precipitation during the growing season (May-September) from 2014 to 2018.**


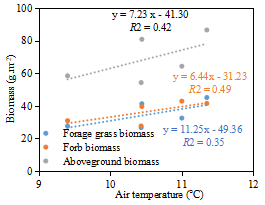

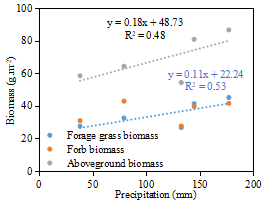


**Fig.** **S2. The correlations between air temperature and precipitation and biomass and CO_2_ fluxes during the plant growing season (May-September) from 2014 to 2018.**

**Fig. S3. Patterns in the plant functional groups composition from 2014 to 2018. The error bars represent standard error (SE). N0-N40: expressed as control, 0 kg, 7 kg, 20 kg, and 40 kg N ha^-1^yr^-1^. Different small letters indicates significant differences (*P* <0.05) among different N addition levels**

**Figure S4. A *priori* structural equation models for effects of N addition on soil availability N and functional group biomass, ecosystem CO_2_ exchange (ER and NEE), and revealed the direct and indirect influences of soil and community characteristics on the effect of NEE and ER to N deposition in the Tibetan Plateau alpine grasslands.**

**N addition can increase soil nutrient availability (path 4, table 1, Fig. 1) and alter community assembly by increasing forage grass and forb biomass (path 1, 2, table 2, Fig. 2, Bai et al., 2008; Yang et al., 2012; Xu et al., 2014). N addition induced change of soil available N content can increase directly** **forage grass and forb biomass by changing of plant height and coverage (path 7, 10, Bai et al., 2008; Yang et al., 2012; Xu et al., 2014). Optimal community assembly can advance ER and NEE (path 9, 11, 14, 15, Duprè et al., 2010; Brueck et al., 2010; Jiang et al., 2012), for example, the increase in forb biomass can affect the ER (path 11, Ammann et al., 2007), and the increase of forage grass biomass can promote NEE (path 16; Yan et al., 2014, Niu et al., 2009; Wang et al., 2015; Bassin et al., 2007). Changes in soil factors then subsequently alter ER and NEE (path 6, 12; Peng et al., 2015). Moreover, N addition can induce many changes, such as changes of plant interactions, which can also alter ER and NEE. Therefore, we separately set up the path from treatments and soil and plant community assembly to ER and NEE.**

**Fig. S5. Seasonal variations of the NEE, ER, and GEP at each measurement day during the five studied years**

**Table S1 Biomass estimation models for different growth forms in each year. *B* indicates biomass (g m^-2^), *H* indicates height (cm), and *C* indicates cover (%).**

| Year | Functional group | Equation | *p* | *n* |
| --- | --- | --- | --- | --- |
| 2014 | Forage grass | *B* = 1.282H +0.059C + 0.585 | <0.001 | 20 |
|  | Forb | *B* = 0.023H +0.312C + 2.257 | <0.001 | 20 |
| 2015 | Forage grass | *B*= 4.772H +0.408C-7.202 | <0.001 | 20 |
|  | Forb | *B*= 9.057H +0.82C-11.57 | <0.001 | 20 |
| 2016 | Forage grass | *B*= 9.946H +0.251C-20.441 | <0.001 | 20 |
|  | Forb | *B*= 3.048H +1.772C-12.815 | <0.001 | 20 |
| 2017 | Forage grass | *B*= 4.919H +1.23C-45.28 | <0.001 | 20 |
|  | Forb | *B*= 8.954H +1.635C-11.967 | <0.001 | 20 |
| 2018 | Sedge | *B* = 9.325H +0.629C-16.889 | <0.001 | 20 |
|  | Gramineae | *B*= 2.265H +1.081C-10.157 | <0.001 | 20 |
|  | Forb | *B* = 8.805H +0.963C-10.29 | <0.001 | 20 |
|  | Leguminous | *B*= 5.166H +1.435C-7.33 | <0.001 | 20 |

**Table S2. Results of repeated measures analysis of variance (RMANOVA) on the effects of N addition (N), year, and their interactions on plant functional groups composition and diversity indices. The values of *P* < 0.05 are in bold**

| Factors | | Sedge traits | | | | Forb traits | | | | Gramineae traits | | | | Diversity index | | | | Community composition | | | | | |
| --- | --- | --- | --- | --- | --- | --- | --- | --- | --- | --- | --- | --- | --- | --- | --- | --- | --- | --- | --- | --- | --- | --- | --- |
|  | | Coverage (%) | | Height (cm) | | Coverage (%) | | Height (cm) | | Coverage (%) | | Height (cm) | | Simpson | | Shannon-Wiener | | Sedge dominance | | Gramineae dominance | | Forb dominance | |
| Factors | df | F value | P value | F value | P value | F value | P value | F value | P value | F value | P value | F value | P value | F value | P value | F value | P value | F value | P value | F value | P value | F value | P value |
| Year | 4 | 17.195 | **<0.001** | 8.533 | **<0.001** | 3.359 | **0.015** | 9.391 | **<0.001** | 15.478 | **<0.001** | 10.584 | **<0.001** | 11.161 | **<0.001** | 8.009 | **<0.001** | 13.283 | **<0.001** | 2.877 | **0.030** | 8.130 | **<0.001** |
| N | 3 | 27.356 | **<0.001** | 18.786 | **<0.001** | 3.386 | **0.024** | 7.350 | **<0.001** | 9.076 | **<0.001** | 9.334 | **0.001** | 9.060 | **<0.001** | 12.702 | **<0.001** | 2.199 | 0.097 | 4.175 | **0.009** | 7.694 | **0.001** |
| Year*N | 12 | 2.075 | **0.032** | 3.969 | **<0.001** | 1.053 | 0.415 | 2.504 | **0.010** | 0.697 | 0.748 | 3.518 | **0.001** | 0.512 | 0.899 | 1.178 | 0.319 | 1.003 | 0.457 | 0.407 | 0.955 | 0.507 | 0.902 |

**Table S3. Patterns of coverage and height in sedge, Gramineae, forb with increasing N addition from 2014 to 2018. Different small letters indicated significant differences (*P* <0.05) among different treatments in the same year.**

| Year | Plots | Coverage of sedge (%) | Height of sedge (cm) | Coverage of Gramineae (%) | Height of Gramineae (cm) | Coverage of forb (%) | Height of forb (cm) |
| --- | --- | --- | --- | --- | --- | --- | --- |
| 2014 | N0 | 21.48±2.39b | 2.52±0.28a | 2.42±0.46a | 4.55±1.35a | 9.53±2.52a | 1.50±0.18a |
|  | N7 | 33±2.26a | 2.74±0.31a | 4.45±3.54a | 3.56±0.38a | 19.73±5.55a | 1.56±0.35a |
|  | N20 | 36.05±2.53a | 2.61±0.28a | 1.33±0.23a | 3.96±0.31a | 19.08±3.47a | 1.16±0.34a |
|  | N40 | 27.83±1.70ab | 2.04±0.13a | 1.65±0.64a | 4.21±0.40a | 11.1±3.59a | 1.04±0.13a |
| 2015 | N0 | 14.75±2.63c | 1.94±0.29b | 4.03±0.41b | 5.17±0.24a | 24.3±0.87b | 1.33±0.13a |
|  | N7 | 24.8±6.36bc | 2.64±0.28ab | 3.43±1.12b | 5.07±0.63a | 34.7±2.84ab | 1.51±0.27a |
|  | N20 | 35±6.88ab | 3.16±0.38a | 8.38±3.85ab | 5.77±0.70a | 42.88±5.33a | 1.36±0.15a |
|  | N40 | 40.93±9.54a | 2.95±0.23a | 10.63±3.86a | 7.38±1.26a | 39.78±2.71a | 1.75±0.09a |
| 2016 | N0 | 37.25±2.03c | 2.31±0.28c | 2.38±0.58a | 4.64±0.46bc | 18.03±2.68a | 1.70±0.08bc |
|  | N7 | 41.63±1.41bc | 2.76±0.23c | 2.38±0.99a | 3.69±1.28c | 33.08±3.48a | 1.52±0.19c |
|  | N20 | 48.38±3.88b | 3.88±0.39b | 3.95±0.37a | 6.91±0.24b | 30.28±1.74a | 2.22±0.13ab |
|  | N40 | 61.95±2.05a | 5.13±0.40a | 5.4±2.58a | 10.13±1.50a | 22.83±2.83ab | 2.84±0.10a |
| 2017 | N0 | 21.88±1.55b | 1.93±0.10c | 1.15±0.21b | 5.15±1.58b | 12.01±1.3a | 1.10±0.09c |
|  | N7 | 30.76±2.63ab | 2.89±0.22b | 1.75±0.37b | 9.75±1.64a | 20.73±3.53a | 2.31±0.29b |
|  | N20 | 37.68±1.85a | 2.94±0.36b | 1.58±0.09b | 8.68±0.28a | 20.81±3.69a | 1.73±0.09bc |
|  | N40 | 38.88±2.97a | 4.08±0.21a | 6.89±2.27a | 8.63±0.55a | 23.24±8.46a | 3.28±0.69a |
| 2018 | N0 | 26.8±1.27b | 2.84±0.42b | 4.38±1.75a | 5.86±0.37ab | 19.88±2.99b | 1.88±0.10a |
|  | N7 | 31.3±1.27b | 2.95±0.25b | 2.95±1.09a | 4.52±1.51b | 28.25±6.73ab | 2.16±0.14a |
|  | N20 | 34.88±2.24b | 3.39±0.41ab | 4.78±0.88a | 6.18±0.31ab | 31.5±5.08a | 2.09±0.31a |
|  | N40 | 51.8±1.68a | 4.14±0.26a | 5.88±1.66a | 7.37±0.40a | 28.85±2.46ab | 2.52±0.07a |

**Table S4. Model selection procedure and statistics for structural equation model explaining NEE. The best model was marked in bold.**

|  | Stepwise removal of non-significant paths | *Df* | *AIC* | *χ^2^* | *P* | *CFI* | *TLI* | *RMSEA* |
| --- | --- | --- | --- | --- | --- | --- | --- | --- |
| *A priori* model |  | 0 | 54.000 | 0.0 |  | 1.000 |  | 0.237 |
| Model 1 | Soil available N🡪 forb biomass | 1 | 52.172 | 0.172 | 0.678 | 1.000 | 1.178 | <0.001 |
| Model 2 | Soil available N🡪 forage grass biomass | 2 | 51.704 | 1.704 | 0.427 | 1.000 | 1.032 | <0.001 |
| **Model 3** | **N addition 🡪 ER** | **3** | **50.655** | **2.655** | **0.448** | **1.000** | **1.025** | **<0.001** |
| Model 4 | N addition 🡪 NEE | 4 | 58.274 | 12.274 | 0.015 | 0.915 | 0.555 | 0.158 |

**Reference**

Bai, Y., Wu, J., Xing, Q., Pan, Q., Huang, J., Yang, D., Han, X., 2008. Primary production and rain use efficiency across a precipitation gradient on the Mongolia plateau. Ecology. 89: 2140–2153

Yang, H.J., Jiang, L., Li, L.H., Li, A., Wu, M.Y., Wan S.Q., 2012. Diversity dependent stability under mowing and nutrient addition: evidence from a 7-year grassland experiment. Ecol Lett. 15: 619–626.

Xu, Z., Ren, H., Cai, J., Wang, R., Li, M.H., Wan, S., Han, X., Lewis, B.J., Jiang, Y., 2014. Effects of experimentally-enhanced precipitation and nitrogen on resistance, recovery and resilience of a semi-arid grassland after drought. Oecologia. 176: 1187–1197.

Duprè C, Stevens CJ, Ranke T, Bleeker A, Peppler-Lisbach C, Gowing, D.G.J., Dise, N.B., Dorland, E., Bobbink, R., Diekmann, M., 2010. Changes in species richness and composition in European acidic grasslands over the past 70 years: the contribution of cumulative atmospheric nitrogen deposition. Global Change Biol. 16: 344–357.

Brueck. H., Erdle, K., Gao, Y., Giese, M., Zhao, Y., Peth, S., 2010. Effects of N and water supply on water use efficiency of a semiarid grassland in Inner Mongolia. Plant Soil. 328: 495–505.

Jiang, L., Guo, R., Zhu, T., Niu, X., Guo, J., Sun, W., 2012. Water- and plant-mediated responses of ecosystem carbon fluxes to warming and nitrogen addition on the Songnen grassland in northeast China. Plos One. 2012; 7: e45205.

Niu, S.L., Yang, H.J., Zhang, Z., Wu, M.Y., Lu, Q., Li, L.H., Han, X.G, Wan, S.Q., 2009. Non-additive effects of water and nitrogen addition on ecosystem carbon exchange in a temperate steppe. Ecosystems. 12: 915–926.

Wang, Y., Jiang, Q., Yang, Z., Sun, W., Wang, D., 2015. Effects of Water and Nitrogen Addition on Ecosystem Carbon Exchange in a Meadow Steppe. PLoS One. 10(5): e0127695.

Bassin, S., Volk, M., Suter, M., Buchmann, N., Jürg Fuhrer., 2007. Nitrogen deposition but not ozone affects productivity and community composition of subalpine grassland after 3yr of treatment. New Phytol. 175(3), 523–534.

Peng, F., You, Q.G., Xu, M.H., Zhou, X.H., Wang, T., Guo, J., 2015. Effects of experimental warming on soil respiration and its components in an alpine meadow in the permafrost region of the Qinghai-Tibet Plateau. Eur. J. Soil Sci., 66, 145-154
